# Pyroglutamate-Modified Amyloid Beta Peptides: Emerging Targets for Alzheimer´s Disease Immunotherapy

**DOI:** 10.2174/1570159X11311050004

**Published:** 2013-09

**Authors:** Roxanna Perez-Garmendia, Goar Gevorkian

**Affiliations:** Instituto de Investigaciones Biomedicas, Universidad Nacional Autonoma de Mexico (UNAM), Mexico DF, Mexico

**Keywords:** Alzheimer´s disease, amyloid-beta, glutaminyl cyclase, immunotherapy, N-terminal truncated amyloid beta, pyroglutamate-modified amyloid-beta.

## Abstract

Extracellular and intraneuronal accumulation of amyloid-beta (Aβ) peptide aggregates in the brain has been hypothesized to play an important role in the neuropathology of Alzheimer’s Disease (AD). The main Aβ variants detected in the human brain are Aβ1-40 and Aβ1-42, however a significant proportion of AD brain Aβ consists also of N-terminal truncated species. Pyroglutamate-modified Aβ peptides have been demonstrated to be the predominant components among all N-terminal truncated Aβ species in AD brains and represent highly desirable and abundant therapeutic targets. The current review describes the properties and localization of two pyroglutamate-modified Aβ peptides, AβN3(pE) and AβN11(pE), in the brain. The role of glutaminyl cyclase (QC) in the formation of these peptides is also addressed. In addition, two potential therapeutic strategies, the inhibition of QC and immunotherapy approaches, and clinical trials aimed to target these important pathological Aβ species are reviewed.

## INTRODUCTION

Extracellular and intraneuronal accumulation of amyloid-beta (Aβ) peptide aggregates in the brain has been hypothesized to play an important role in the neuropathology of Alzheimer’s Disease (AD) [1-7]. Aβ is generated from the amyloid precursor protein (APP) by the sequential proteolysis by the β-secretase activity (cysteine proteases and β-site APP-cleaving enzyme (BACE)) and by γ-secretase (a multimeric protein complex composed of presenilin, nicastrin, Aph-1 and Pen-2), and is secreted from cells of neuronal origin *via* major regulated as well as minor constitutive secretory pathway [8-10]. Aβ is a normal product of cell metabolism and is present in the plasma and in cerebrospinal fluid (CSF) in healthy individuals. However, abnormal and excessive accumulation of Aβ in the brain leads to the formation of toxic Aβ aggregates that induce synaptic dysfunction and neuronal loss [2,3]. 

The main Aβ variants detected in the human brain are Aβ1-40 and Aβ1-42, however a significant proportion of AD brain Aβ consists also of N-terminal truncated species (Aβn-40/42 where n=2 to 11) [1, 11-22]. Most of N-truncated Aβ peptides have been considered to be the degradation products of full-length Aβ; however, the overexpression of BACE in cultured cells led to the conclusion that Aβ11-40/42 may be generated intracellularly directly from APP by BACE proteolysis at an alternative site, between Tyr^10^ and Glu^11^, depending on enzyme’s precise localization within the cell, being the endoplasmic reticulum the preferred site of full-length Aβ production whereas truncated Aβ is formed in trans-Golgi network [8,23-25]. 

Previous reports demonstrated that amyloid aggregates in AD brain and in brain of cognitively normal elderly subjects have different composition and that the toxic effect of these aggregates is correlated with the predominance of the N-terminal truncated species over the full length Aβ [25-27]. Pyroglutamate-modified Aβ peptides have been demonstrated to be the predominant components among all N-terminal truncated Aβ species in AD brain [28-31]. AβN3(pE), Aβ peptide bearing amino-terminal pyroglutamate at position 3, has been shown to be a major N-truncated/modified constituent of intracellular, extracellular and vascular Aβ deposits in AD brain tissue [14,15,17,28,32-35]. Importantly, it has been demonstrated that AβN3(pE) progressively accumulates in the brain at the earliest stages of AD even before the appearance of clinical symptoms suggesting that this peptide is a potential seeding specie and may play an important role in the formation of pathological amyloid aggregates [14,36]. 

Previous studies have also demonstrated that shortened/modified Aβ forms are significantly more resistant to degradation, aggregate more rapidly *in vitro* and exhibit similar or, in some cases, increased toxicity in neuronal cultures compared to the full-length peptides [37-44]. In addition, 12-weeks-old wild type mice (C57BL/6 strain) displayed impaired spatial working memory and delayed memory acquisition in Y-maze and Morris water maze tests after intracerebroventricular injection of aggregated AβN3(pE) [41]. 

Thus, the N-terminally truncated/modified Aβ peptides represent highly desirable and abundant therapeutic targets [30, 35]. 

## PYROGLUTAMATE-MODIFIED AΒ PEPTIDES

Although previous studies pointed to the modifications in the NH2 terminus of Aβ peptides isolated from the AD brain, making them resistant to Edman degradation [45, 46], it was in 1992 when Mori and collaborators demonstrated for the first time the presence of AβN3(pE) in brain samples from patients that had neuropathologically typical AD [11]. Soon after, other groups detected two N-truncated/pyroglutamate-modified peptides, AβN3(pE) and AβN11(pE), in amyloid plaques in AD brain [13-15, 31, 47, 48]. In these studies, both peptides were shown to form the central core of amyloid aggregates pointing to the hypothesis that their deposition in AD brain may have preceded that of the full length Aβ peptide [14, 15]. This is not surprising because AβN3(pE) as well as AβN11(pE) are expected to be more hydrophobic owing to the loss of three charges in AβN3(pE) and 6 charges in AβN11(pE), and are also expected to have longer life than full length Aβ since common aminopeptidases would not digest them and pyroglutamate-specific amino-peptidases are required. 

Diffuse amyloid plaques, one of the earliest forms of amyloid deposition, have also been shown to contain AβN3(pE) peptide [49]. In addition, analysis of water-soluble amyloid peptides, that are thought to precede amyloid plaques in AD brain, revealed that AβN3(pE) is the most abundant form among Aβ species [17]. Interestingly, AβN11(pE) was the main peptide detected in cotton wool plaques, a round lesion that lacks a central amyloid core, in individuals affected by early-onset familial AD associated with mutations in the presenilin 1 [21]. AβN3(pE) was present in these samples in less amounts [21]. 

AβN3(pE) was also detected in vascular amyloid deposits although in relatively minor quantities. Thus, Kuo and collaborators have demonstrated that neuritic plaques from individuals with AD had about 51% of AβN3(pE) while vascular amyloid from these individuals contained an average of 11% of AβN3(pE) [50]. 

Recently, peri-synaptic discrete and granular AβN3(pE) aggregates that co-localized with the presynaptic protein synaptophysin were detected in the postmortem brain samples from individuals with early stages of AD suggesting that they may contribute to early cognitive dysfunction [51]. Importantly, intraneuronal AβN3(pE) oligomers were shown to represent an important pathological intermediate appearing at a time point when behavioral deficits occur [52]. Finally, De Kimpe and collaborators demonstrated that in postmortem human brain tissue, aggregated AβN3(pE) is predominantly found in the lysosomes of both neurons and glial cells and that intracellular AβN3(pE) amount increases with age [53]. 

Studies on biophysical properties of pyroglutamate-modified Aβ peptides demonstrated that they form β-sheet structure more readily than the corresponding full-length peptides suggesting them to be potential seeding species of aggregate formation [38, 40, 54]. Sedimentation experiments showed that the pyroglutamate-containing Aβ peptides have greater aggregation propensities than the corresponding full-length peptides [38]. A detailed kinetic and structural study of full length Aβ1-42 and two pyroglutamate species, AβN3(pE) and AβN11(pE), performed by two complementary and independent techniques, circular dichroism and Thioflavine T fluorescence spectroscopy, together with electron microscopy revealed that AβN3(pE) shows substantially faster aggregation kinetics compared with full length peptide [42]. Interestingly, transmission electron microscopy analysis revealed that AβN3(pE) has an inhibitory effect on full length Aβ1-42 fibrillogenesis, probably maintaining the peptides in oligomeric/prefibrillar conformation, that has been demonstrated to be more toxic for the cells and for the progression of AD pathogenesis [7, 42, 55]. Recently, Sun and collaborators performed structural analysis of AβN3(pE) using high-resolution NMR spectroscopy and demonstrated decreased helical propensity in pyroglutamate-modified peptide compared with Aβ40 under exactly same conditions, in agreement with the observation of increased tendency to form β-sheet structures under physiological conditions [44]. 

Studies on cytotoxic properties of pyroglutamate-modified Aβ peptides revealed that AβN3(pE) induced significantly more cell loss than other Aβ species in rat cultured hippocampal neurons and cortical astrocytes [39]. Authors have demonstrated that AβN3(pE) peptides were heavily distributed on plasma membrane and within the cytoplasm of treated cells [39]. In addition, AβN3(pE) was shown to cause DNA fragmentation in cultured neurons but not in cortical astrocytes. In contrast, no LDH release, which indicates membrane damage and lysis of damaged cells, was observed in cultured neurons while LDH amount increased by 32-40% after treatment of astrocytes with AβN3(pE) [39]. These results suggest that the pyroglutamate-modified peptides may share similar degenerative mechanisms, apoptosis in neurons and necrosis in astrocytes, with full length Aβ [39]. The highly pathogenic effect of AβN3(pE) was supported also by the finding that its early aggregates alter the membrane permeability probably by forming membrane pores [26]. In addition, AβN3(pE) was shown to induce a redox-sensitive neuronal apoptosis involving caspase activation and an arachidonic acid-dependent pro-inflammatory pathway in primary neuronal cultures [41]. Recently, our group demonstrated that AβN3(pE) oligomers induce phosphatidylserine externalization and membrane damage in differentiated SH-SY5Y cells [56]. Moreover, AβN3(pE) was shown to inhibit long term potentiation in mouse hippocampal slices [54]. Finally, De Kimpe and collaborators showed that AβN3(pE) oligomers cause lysosomal membrane permeabilization leading to impaired lysosomal function and aberrant exposure of cellular components to lysosomal enzymes [53]. 

In a recent study, Nussbaum and collaborators demonstrated that AβN3(pE) may form low molecular weight hybrid oligomers with the full length Aβ peptide and cause an accelerated misfolding and oligomerization of Aβ1-42 leading to toxic structures that propagate by a prion-like mechanism [43]. These mixed oligomers as well as 100% AβN3(pE) but not 100% Aβ1-42 oligomers potently inhibited long-term potentiation of mouse hippocampal neurons in slice cultures and significantly reduced cell viability as assessed in primary wild-type neuron cultures by XTT assay, although in the latter experiments 100% AβN3(pE) was less toxic compared with hybrid oligomers [43]. 

Importantly, in Tg2576 mice, the most frequently used APP transgenic mouse model during first decade of AD immunotherapy studies, as well as in other commonly used Tg mice, pyroglutamate-modified Aβ peptides were not detected at all or detected in old animals at levels far lower than in human AD brain [27, 57, 58]. Schilling and collaborators detected AβN3(pE) in Tg2576 mice brain at 12 months of age but this Aβ specie still represented only 0.1-0.5% of total Aβ [59]. A few Tg mouse strains (APP/PS1KI, TBA2.1, TBA2.2, TBA42) were developed to produce AβN3(pE) peptide, and the presence of intraneuronal pool as well as extracellular aggregates containing AβN3(pE) have been shown to correlate with the development of early synaptic and behavioral deficits as well as with microgliosis, astrocytosis, hippocampal atrophy and neuronal loss in these mice [34, 60-67]. 

## POSSIBLE TREATMENT STRATEGIES

### Glutaminyl Cyclase Inhibition

**Fig. (1):**
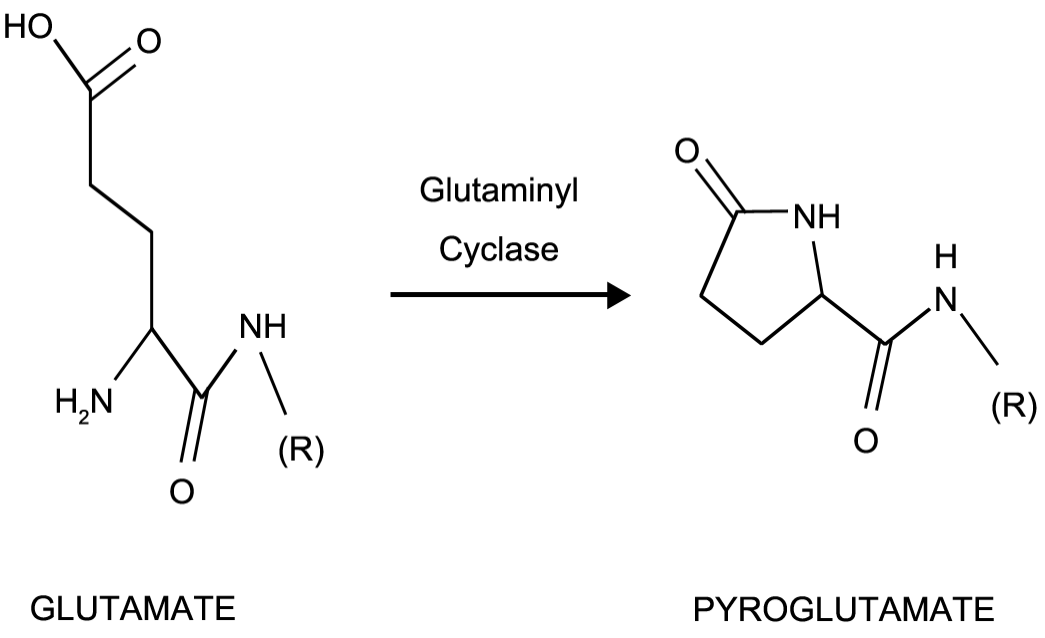
Glutaminyl cyclase (QC) converts N-aminoterminal glutamate residue to pyroglutamate.

Conversion of N-terminal glutamate (Glu^1^) residue to pyroglutamate (Fig. **1**) is catalysed by the glutaminyl cyclase (QC), an enzyme highly abundant in mammalian brain [68]. 

QC has a key function in the posttranslational processing of several hormones, converting amino-terminal glutamine into pyroGlu [69]. It has been demonstrated that *in vitro* amino-terminal glutamate (Glu^1)^ is also converted to pyroGlu by incubation at pH 6.0 in the presence of QC [68]. If QC was boiled before addition, formation of the pyroGlu peptides was negligible; no conversion of glutamate to pyroGlu was detected at basic pH values in contrast to well known glutamine (Gln) modification by QC that occurs at an optimum pH 8.0 [68]. Importantly, both Aβ and QC, have been found to be localized within the acidic secretory vesicles [10, 69]. Interestingly, while co-transfection of APP and QC led to pyroGlu formation in HEK293 cells, the addition of recombinant QC to the same cell culture medium generated only minor amounts of AβN3(pE) suggesting that conversion of Glu^1^ to pyroGlu, at least in this case, is favored intracellularly [70]. Importantly, trans-Golgi network, a mildly acidic (pH 5.9-6.5) organelle, was reported to be a predominant cellular compartment for truncated Aβ11-40/42 production and, in agreement with above mentioned studies, may be also the site of Glu1 conversion into pyroglutamate, resulting in AβN11(pE) [23, 68, 69]. Formation of AβN3(pE) *in vivo/in situ* was observed after microinjection of Aβ1-40 and Aβ3-40 into the rat cortex, and the generation of AβN3(pE) was significantly inhibited by intracortical microinjection of a QC inhibitor [71]. Finally, it has been demonstrated that 5XFAD/hQC bigenic mice, obtained by crossing 5XFAD mice with Tg mice expressing human QC under the control of the Thy1 promoter, showed significant elevation in AβN3(pE) levels and a significant motor and working memory impairment compared with 5XFAD mice [66]. Importantly, QC knock-out rescues the behavioral impairments in 5XFAD mice clearly demonstrating that QC is crucial for modulating AβN3(pE) levels [66]. 

Importantly, glutaminyl cyclase mRNA and protein levels were upregulated in brain samples from individuals with AD compared with samples from normal aging individuals, correlating with significantly larger amount of AβN3(pE) in AD brain detected by ELISA analysis as well as by immunohistochemistry [59, 72]. In the latter study authors demonstrated that disturbed Ca2+ homeostasis results in upregulation of QC in differentiated neuroblastoma cells, suggesting that disruption of Ca2+ homeostasis, one of the early pathogenic factors observed in AD, may contribute to the formation of pyroglutamate-modified Aβ peptides [72]. Recently, higher levels of glutaminyl cyclase mRNA and protein in peripheral blood from AD patients compared with age-matched controls were found, and a correlation between glutaminyl cyclase expression and the severity of dementia was observed [73]. 

Interestingly, different types of AβN3(pE) aggregates, focal and diffuse deposits, were identified in defined layers of the AD hippocampus [74]. The focal/cored AβN3(pE) aggregates were found to be associated with the somata of QC-expressing interneurons or neuronal debris suggesting that AβN3(pE) is produced and deposited intracellularly [74]. In contrast, diffuse AβN3(pE) aggregates were not associated with QC-expressing neurons, and authors hypo-thesize that QC and/or Aβ/AβN3(pE) may be transported from entorhinal cortex projection neurons and released at hippocampal terminal zones forming extracellular deposits; however, diffuse AβN3(pE) deposits could also be localized within neurits of QC-rich neurons arising from entorhinal cortex [74]. These observations indicate that QC may convert Glu^1^ to pyroglutamate in AD both intra- and extracellularly in contrast to results obtained previously in transfected cells [70]. 

One of the possible treatment strategies for AD targeting pyroglutamate-modified Aβ may be, thus, the inhibition of QC, preventing the conversion of glutamate residue to pyroglutamate and the formation of AβN3(pE) and AβN11(pE). Cynis and collaborators demonstrated that the QC-specific inhibitor P150/03 decreases cyclization of glutamate at the N-terminus and formation of AβN3(pE) in cultured mammalian cells [75]. To further confirm the specific effect of QC inhibitor, authors generated transgenic *Drosophila* flies with neuron-specific expression of Aβ1-42 or AβN3(pE) [59]. A four-week treatment of AβN3(pE)-transgenic flies with a QC inhibitor PBD150 led to a significant decrease of AβN3(pE) while total Aβ was not affected in flies expressing Aβ1-42, suggesting that PBD150 specifically reduces AβN3(pE) [59]. Subsequently, QC inhibitor was applied orally to 4-, 6- and 10-month-old APP-Tg mice for 6 to 10 months to study effects of glutamate-pyroglutamate conversion on the concentrations of AβN3(pE) as well as other Aβ species [59]. In all three trials, a dose dependent decrease of cortical plaque formation, plaque-associated inflammation and total Aβ and AβN3(pE) concentrations in the insoluble Aβ pool as well as improved memory were observed [59]. 

Currently, PQ912, a glutaminyl cyclase inhibitor discovered by Probiodrug and shown to be safe and well tolerated in a Phase I clinical trial, is in further clinical development for the treatment of AD (www.probiodrug.de). 

### Immunotherapy

Immunotherapy approaches, both active immunization with Aβ peptide or passive transfer of anti-Aβ antibodies, have been demonstrated to decrease amyloid deposits and associated neuronal and inflammatory pathologies and reverse Aβ-related cognitive deficits in several amyloid precursor protein transgenic (APP/Tg) mouse as well as canine and primates models of AD [76-84]. The majority of these studies used mainly Aβ1-40 or Aβ1-42 as an immunogen for active immunization, which induced antibodies specific for amino-terminal part (EFRH epitope) of Aβ. However, pyroglutamate-modified forms of the Aβ lack this critical B-cell epitope. Nowadays, a number of clinical studies investigating the effectiveness of anti-Aβ immunotherapy in AD patients are underway. Full length Aβ peptide as well as a number of immunogens based on the N-terminal immunodominant epitope are being tested in different elegant strategies. However, these immunization strategies would induce antibodies recognizing the full length Aβ but not necessarily N-truncated modified species. Interestingly, a detailed analysis of the neuropathology and Aβ spectrum in a Bapineuzumab (a humanized antibody raised against the N-terminus of Aβ 1-42) immunotherapy recipient revealed the presence of various Aβ peptides, including AβN3(pE) [85]. In this patient, bapineuzumab immunotherapy neither resulted in detectable clearance of amyloid aggregates nor prevented further cognitive impairment despite promising results with the same antibody in transgenic mice [86, 87]. One of the possible explanations of the failure of bapineuzumab to reduce amyloid aggregates in this case may be the presence of N-truncated/pyroglutamate-modified Aβ. As we discussed above, N-truncation and glutamate to pyroglutamate modification at position 3 or 11 of Aβ may lead to altered secondary and tertiary structures and prevent the recognition by a given antibody. Thus, there is an urgent need to design novel immunotherapy strategies directed against N-truncated/pyroglutamate-modified Aβ peptides and consider them for vaccine development for AD. 

In 2003, Sergeant and collaborators, after a thorough characterization of Aβ aggregates in human brain, concluded that truncated Aβ species are early, pathological and abundant antigens and proposed that they could be an ideal target for vaccination [36]. In recent years, a couple of laboratories started to include pyroglutamate-modified Aβ in their research agenda. Our group performed first studies on immunogenicity of AβN3(pE) and AβN11(pE) in rabbits and demonstrated that while AβN3(pE) peptide induces antibodies specifically binding to AβN3(pE), anti-AβN11(pE) antibodies recognize two pyroglutamate species, AβN3(pE) and AβN11(pE), and full-length Aβ as well [56, 88]. The latter results suggest that AβN3(pE), AβN11(pE) and full-length Aβ may share a common B cell epitope, and are important for designing immunogens capable of inducing antibodies targeting three main pathological species of the Aβ peptide present in human brain. This should significantly enhance the efficacy of immunotherapy in the CNS of AD patients, because only approximately 0.1% of the antibody in the blood gains entry into the brain. We also performed epitope mapping of anti-AβN3(pE) and anti-AβN11(pE) antibodies and demonstrated the presence of one immunodominant epitope at the N-terminal part of AβN3(pE) and two major B cell epitopes in AβN11(pE) (one at the N-terminal part (aa 11-15) and another at the central part (aa 20-24) of the peptide) [56, 88]. Studies on immunogenic properties of identified epitopes/mimotopes are underway.

Wirths and collaborators generated a monoclonal antibody 9D5 selectively binding to low molecular weight AβN3(pE) oligomers and demonstrated that this antibody inhibits aggregation and toxicity of AβN3(pE) *in vitro* and shows a specific staining profile in AD and 5XFAD Tg mice brain [35, 89]. In addition, passive immunization with 9D5 in 5XFAD mice significantly reduced intracellular AβN3(pE) oligomers, general plaque load in hippocampus and cortex as well as behavioral deficits [35]. Authors proposed that intraneuronal AβN3(pE) oligomers represent an important early pathological step and that targeting these toxic aggregates may also have an impact on other pathological Aβ species [35, 52]. 

Lemere and collaborators evaluated in AD-tg mice another monoclonal antibody specifically binding to AβN3(pE) in passive immunization protocols and demonstrated that it is able to lower total Aβ deposition in a prevention trial [90]. Moreover, weekly anti-AβN3(pE) antibody administration for 7 weeks reduced plaque burden in the absence of microhemorrhages in a therapeutic trial in 23-month-old, plaque bearing Tg mice [90]. This study confirmed that selective removal of AβN3(pE) aggregates may lower deposition of multiple Aβ species in the brain [90]. 

Recently, a plaque-specific anti-AβN3(pE) monoclonal antibody mE8 was developed and used for passive immunization in both prevention and therapeutic studies in AD-Tg mice [91]. Interestingly, this antibody reduced existing plaques when applied weekly for 3 months to 23-month-old mice while the well-known 3D6 anti-Aβ monoclonal antibody, the murine equivalent of bapineuzumab used in clinical trials, had no effect in the same immunization protocol [91]. However, behavioral studies were not performed at the end of mE8 immunization to conclude if this antibody would be a suitable immunotherapeutic for AD in future. Quite different results were obtained with mE8 in a prevention study in 5.5-month-old AD-Tg mice, an age before the initiation of robust Aβ deposition. In this study, treatment with 3D6 antibody resulted in a significant decrease of hippocampal Aβ as compared with the control IgG; in contrast, the plaque-specific anti-AβN3(pE) monoclonal antibody mE8 did not show significant reduction in Aβ, suggesting that the mechanism of action of these antibodies is different [91]. Different results observed by Lemere and DeMattos groups [90, 91] in therapeutic studies using anti-AβN3(pE) antibodies may be explained, in part, by the fact that they recognize different amyloid aggregates, and, probably, the effective passive immunization protocol should include more than one anti-Aβ antibody. Importantly, anti-AβN3(pE) antibodies did not exacerbate micro-hemorrhage yet were able to significantly remove existing plaques, while the 3D6 antibody induced an increase in microhemorrhage and did not remove plaques [91]. These results clearly demonstrated that amyloid plaques can be removed without this important adverse event if suitable immunotherapy approach is applied. 

To the best of our knowledge, there is only one clinical trial evaluating active immunotherapy approach targeting pyroglutamate-modified Aβ. Affiris AG developed a new immunogen AFFITOPE AD-03 based on mimotopes and started in 2010 a phase I trial to assess its tolerability and safety after repeated subcutaneous administration with or without an adjuvant (alum) in patients with mild to moderate AD (http://www.clinicaltrials.gov). In December 2011, authors reported that AFFITOPE AD-03 passed successfully phase I clinical testing. This announcement is very promising although alum as an adjuvant is not the most suitable choice for AD patients because its’ known neurotoxic effect [92]. 

Solanezumab, a monoclonal humanized anti-Aβ central region antibody is currently being tested in clinical trials in passive immunization protocols in patients with mild to moderate AD [93-96]. This antibody was well tolerated after single or multiple weekly doses up to 400mg and no evidence of meningoencephalitis, microhemorrhage or vasogenic edema was present in patients, but no changes in cognitive scores occurred [93, 95]. However, the first comprehensive analysis of two Phase III clinical trials performed mainly in the Americas, Western Europe, Australia and Japan demonstrated that monthly administration of solanezumab during 18 months had although small but real beneficial cognitive effect (http://www.alzforum.org/new/detail.asp?id=3288). Since this antibody binds to a central region of Aβ, it may recognize N-truncated species too. However, N-truncation/modification of the molecule may change exposed epitopes, and antibodies raised against pyroglutamate-modified Aβ species are highly warranted. Ideally, a panel of anti-Aβ antibodies should be evaluated for developing effective immunotherapy approach for AD. 

## CONCLUSION

The main Aβ variants detected in the human brain are Aβ1-40 and Aβ1-42, however a significant proportion of AD brain Aβ consists also of N-terminal truncated species. Pyroglutamate-modified Aβ peptides have been demonstrated to be the predominant components among all N-terminal truncated Aβ species. Importantly, it has been demonstrated that these peptides progressively accumulate in the brain at the earliest stages of AD even before the appearance of clinical symptoms, pointing to the hypothesis that they are potential seeding species and may play an important role in the formation of pathological amyloid aggregates. For this reason, N-terminal truncated/pyroglutamate-modified Aβ peptides represent highly desirable and abundant therapeutic targets. 

One of the possible treatment strategies for AD targeting pyroglutamate-modified Aβ may be the inhibition of QC, preventing the conversion of glutamate residue to pyroglutamate and the formation of AβN3(pE) and AβN11(pE). Although a glutaminyl cyclase inhibitor discovered by Probiodrug and shown to be safe and well tolerated in a Phase I clinical trial, is now in further clinical development for the treatment of AD, this strategy has a drawback of inhibiting an enzyme with a key function in the posttranslational processing of several hormones, converting amino-terminal glutamine into pyroGlu. Thus, alternative approaches targeting pyroglutamate-modified Aβ are warranted.

The majority of concluded and ongoing immunotherapy studies for AD used mainly the full length Aβ1-40 or Aβ1-42 as an immunogen for active immunization, inducing antibodies specific for amino-terminal part (EFRH epitope) of the Aβ. However, pyroglutamate-modified forms of the Aβ lack this critical B-cell epitope and consequently, would not be targeted in these studies. Thus, there is an urgent need to design novel immunotherapy strategies directed against N-truncated/pyroglutamate-modified Aβ peptides and consider them for vaccine development for AD. Few laboratories are currently evaluating new therapeutic and preventive immunization protocols using anti-AβN3(pE) antibodies. Also, research on antigenic and immunogenic properties of both pyroglutamate-modified/N-truncated Aβ peptides is underway. These studies may provide promising diagnostic and therapeutic tools, targeting all pathological amyloid species involved in AD in the future. 

## References

[1] Masters CL, Simms G, Weinmann NA, Multhaup G, McDonald BL, Beyreuther K (1985). Amyloid plaque core protein in Alzheimer disease and Down syndrome. Proc. Natl. Acad. Sci. U.S.A.

[2] Walsh DM, Selkoe DJ (2004). Deciphering the molecular basis of memory failure in Alzheimer's diseas. Neuron.

[3] Haass C, Selkoe DJ (2007). Soluble protein oligomers in neurodegeneration lessons from the Alzheimer’s amyloid beta-peptide. Nat. Rev. Mol. Cell. Biol.

[4] LaFerla F, Green KN, Oddo S (2007). Intracellular amyloid-ß in Alzheimer's disease. Nat. Rev. Neurosci.

[5] Gouras GK, Tampellini D, Takahashi RH, Capetillo-Zarate E (2010). Intraneuronal beta-amyloid accumulation and synapse pathology in Alzheimer’s disease. Acta Neuropathol.

[6] Ballard C, Gauthier S, Corbett A, Brayne C, Aarsland D, Jones E (2011). Alzheimer´s disease. The Lancet.

[7] Ferreira ST, Klein WL (2011). The Aß oligomer hypothesis for synapse failure and memory loss in Alzheimer’s disease. Neurobiol. Learn. Mem.

[8] Vassar R, Bennet BD, Babu-Khan S, Khan S, Mendiaz EA, Denis P, Teplow DB, Ross S, Amarante P, Loeloff R, Luo Y, Fisher S, Fuller J, Edenson S, Lile J, Jarosinski MA, Biere AL, Curran E, Burgess T, Louis JC, Collins F, Treanor J, Rogers G, Citron M (1999). Beta-secretase cleavage of Alzheimer´s amyloid precursor protein by the transmembrane aspartic protease BACE. Science.

[9] Selkoe DJ (2001). Alzheimer´s disease genes proteins and therapy. Physiol. Rev.

[10] Hook VY, Reisine T D (2003). Cysteine proteases are the major beta-secretase in the regulated secretory pathway that provides most of the beta-amyloid in Alzheimer´s disease role of BACE1 in the constitutive secretory pathway. J. Neurosci. Res.

[11] Mori H, Takio K, Ogawara M, Selkoe D J (1992). Mass spectrometry of purified amyloid beta protein in Alzheimer's disease. J. Biol. Chem.

[12] Seubert P, Vigo-Pelfrey C, Esch F, Lee M, Dovey H, Davis D, Sinha S, Schiossmacher M, Whaley J, Swindlehurst C, McCormack R, Wolfert R, Selkoe D, Lieberburg I, Schenk D (1992). Isolation and quantification of soluble Alzheimer’s beta-peptide from biological fluids. Nature.

[13] Miller D L, Papayannopoulos I A, Styles J, Bobin S A, Lin Y Y, Biemann K, Iqbal K (1993). Peptide compositions of the cerebrovascular and senile plaque core amyloid deposits of Alzheimer´s disease. Arch. Biochem. Biophys.

[14] Saido T C, Iwatsubo T, Mann DMA, Shimada H, Ihara Y, Kawashima S (1995). Dominant and differential deposition of distinct ß-amyloid peptide species AßN3(pE) in senile plaques. Neuron.

[15] Saido T C, Yamao-Harigaya W, Iwatsubo T, Kawashima S (1996). Amino- and carboxyl-terminal heterogeneity of ß-amyloid peptides deposited in human brain. Neurosci. Lett.

[16] Naslund J, Karlstrom A R, Tjernberg L O, Schierhorn A, Terenius L, Nordstedt C (1996). High-resolution separation of amyloid beta-peptides structural variants present in Alzheimer´s disease amyloid. J. Neurochem.

[17] Russo C, Saido T C, DeBusk L M, Tabaton M, Gambetti P, Teller J K (1997). Heterogeneity of water-soluble amyloid beta-peptide in Alzheimer´s disease and Down´s syndrome brains. FEBS Lett.

[18] Tekirian T L, Markesbery W R, Russel M J, Wekstein D R, Patel E, Geddes J W (1998). N-terminal heterogeneity of parenchymal and cerebrovascular Abeta deposits. J. Neuropathol. Exp. Neurol.

[19] Larner A J (1999). Hypothesis amyloid ß-peptides truncated at the N-terminus contribute to the pathogenesis of Alzheimer´s disease. Neurobiol. Aging.

[20] Kumar-Singh S, DeJonghe C, Cruts M, Kleinert R, Wang R, Mercken M, DeStrooper B, Vanderstichele H, Lofgren A, Vanderhoevens H, Vanmechelen E, Kroisel PM, VanBroeckhoven C (2000). Nonfibrillar diffuse amyloid deposition due to a gamma(42)-secretase site mutation points to an essential role for N-truncated A beta(42) in Alzheimer´s disease. Hum. Mol. Genet.

[21] Miravalle L, Calero M, Takao M, Roher A E, Ghetti B, Vidal R (2005). Amino-terminally truncated Aß peptide species are the main component of cotton wool plaques. Biochemistry.

[22] Vanderstichele H, DeMeyer G, Andreasen N, Kostanjevecki V, Wallin A, Olsson A, Blennow K, Vanmechelen E (2005). Amino-truncated ß-amyloid 42 peptides in cerebrospinal fluid and prediction of progression of mild cognitive impairment. Clin. Chem.

[23] Huse J T, Liu K, Pijak D S, Carlin D, Lee VMY, Doms RW (2002). ß-Secretase processing in the trans-Golgi network preferentially generates truncated amyloid species that accumulate in Alzheimer’s disease brain. J. Biol. Chem.

[24] Lee EB, Skovronsky DM, Abtahian F, Doms RW, Lee VMY (2003). Secretion and intracellular generation of truncated Aß in ß-site amyloid-ß precursor protein-cleaving enzyme expressing human neurons. J. Biol. Chem.

[25] Liu K, Solano I, Mann D, Lemere C, Mercken M, Trojanowski JQ, Lee VMY (2006). Characterization of Aß11-40/42 peptide deposition in Alzheimer’s disease and young Down’s syndrome brains implication of N-terminally truncated Aß species in the pathogenesis of Alzheimer’s disease. Acta Neuropathol.

[26] Piccini A, Russo C, Gliozzi A, Relini A, Vitali A, Borghi R, Giliberto L, Armirotti A, D’Arrigo C, Bachi A, Cattaneo A, Canale C, Torrassa S, Saido T C, Markesbery W, Gambetti P, Tabaton M (2005). ß-Amyloid Is Different in Normal Aging and in Alzheimer Disease. J. Biol. Chem.

[27] Guntert A, Dobeli H, Bohrmann B (2006). High sensitivity analysis of amyloid-beta peptide composition in amyloid deposits from human and PS2APP mouse brain. Neuroscience.

[28] Hosoda R, Saido T C, Otvos LJr, Arai T, Mann DM, Lee VM, Trojanowski JQ, Iwatsubo T (1998). Quantification of modified amyloid beta peptides in Alzheimer disease and Down syndrome brains. J. Neuropathol. Exp. Neurol.

[29] Gunn AP, Masters CL, Cherny RA (2010). Pyroglutamate-Aß: role in the natural history of Alzheimer´s disease. Int. J. Biochem. Cell Biol.

[30] Jawhar S, Wirths O, Bayer T A (2011). Pyroglutamate amyloid ß (Aß) a hatchet man in Alzheimer Disease. J. Biol. Chem.

[31] Sullivan CP, Berg EA, Elliot-Bryant R, Fishman JB, McKee AC, Morin PJ, Shia MA, Fine RE (2011). Pyroglutamate-Aß 3 and 11 colocalize in amyloid plaques in Alzheimer´s disease cerebral cortex with pyroglutamate-Aß 11 forming central core. Neurosci. Lett.

[32] Harigaya Y, Saido T C, Eckman C B, Prada C M, Shoji M, Younkin S G (2000). Amyloid ß protein starting pyroglutamate at position 3 is a major component of the amyloid deposits in the Alzheimers disease brain. Biochem. Biophys. Res. Comm.

[33] Portelius E, Bogdanovic N, Gustavsson M K, Volkmann I, Brinkmalm G, Zetterberg H, Winblad B, Blennow K (2010). Mass spectrometric characterization of brain amyloid beta isoform signatures in familial and sporadic Alzheimer´s disease. Acta Neuropathol.

[34] Wirths O, Bethge T, Marcello A, Harmeier A, Jawhar S, Lucassen P J, Milthaup G (2010). Pyroglutamate Abeta pathology in APP/PS1KI mice sporadic and familial Alzheimer’s disease cases. J. Neural. Transm.

[35] Wirths O, Erck C, Martens H, Harmeier A, Geumann C, Jawhar S, Kumar S, Multhaup G, Walter J, Ingelsson M, Degerman-Gunnarsson M, Kalimo H, Huitinga I, Lannfelt L, Bayer T A (2010). Identification of low molecular weight pyroglutamate Aß oligomers in Alzheimer Disiease.A novel tool for therapy and diagnosis. J. Biol. Chem.

[36] Sergeant N, Bombois S, Ghestem A, Drobecq H, Kostanjevecki V, Missiaen C, Wattez A, David J P, Vanmechelen E, Sergheraert C, Delacourte A (2003). Truncated beta-amyloid peptide species in pre-clinical Alzheimer´s disease as new targets for the vaccination approach. J. Neurochem.

[37] Pike CJ, Overman MJ, Cotman CW (1995). Amino-terminal Deletions Enhance Aggregation of beta-Amyloid Peptides in vitro. J. Biol. Chem.

[38] He W, Barrow CJ (1999). The A beta 3-pyroglutamyl and 11-pyroglutamyl peptides found in senile plaque have greater beta-sheet forming and aggregation propensities in vitro than full-length A beta. Biochemistry.

[39] Russo C, Violani E, Salis S, Venezia V, Dolcini V, Damonte G, Benatti U, D’Ariigo C, Patrone E, Carlo P, Schettini G (2002). Pyrglutamate-modified amyloid ß-peptides – AßN3(pE) – strongly affect cultured neuron and astrocyte survival. J. Neurochem.

[40] Schilling S, Lauber T, Schaupp M, Manhart S, Scheel E, Bohm G, Demuth H U (2006). On the seeding and oligomerization of pGlu-amyloid peptides (in vitro). Biochemistry.

[41] Youssef I, Florent-Bechard S, Malaplate-Armand C, Koziel V, Bihain B, Olivier J L, Leininger-Muller B, Kriem B, Oster T, Pillot T (2007). N-truncated amyloid-ß oligomers induce learning impairment and neuronal apoptosis. Neurobiol. Aging.

[42] D’Arrigo C, Tabaton M, Perico A (2009). N-terminal truncated pyroglutamyl ß amyloid peptide Aßpy3-42 shows a faster aggregation kinetics than the full-length Aß1-42. Biopolymers.

[43] Nussbaum J M, Schilling S, Cynis H, Silva A, Swanson E, Wangsanut T, Tayler K, Wiltgen B, Hatami A, Ronicke R, Reymann K, Hutter-Paier B, Alexandru A, Jagla W, Graubner S, Glabe CG, Demuth HU, Bloom GS (2012). Prion-like behaviour and tau-dependent cytotoxicity of pyroglutamylated amyloid-ß. Nature.

[44] Sun N, Hartmann R, Lecher J, Stoldt M, Funke SA, Gremer L, Ludwig HH, Demuth HU, Kleinschmidt M, Willbold D (2012). Structural analysis of the pyroglutamate-modified isoform of the Alzheimer´s disease-related amyloid-ß using NMR stectroscopy. J. Pept. Sci.

[45] Selkoe DJ, Abraham CR, Podlisny MB, Duffy LK (1986). Isolation of low-molecular-weight proteins from amyloid plaque fibers in Alzheimer’s disease. J. Neurochem.

[46] Miller DL, Currie JR, Iqbal K, Potempska A, Styles J (1989). Relationships among the cerebral amyloid peptides and their precursors. Ann. Med.

[47] Naslund J, Schierhorn A, Hellman U, Lannfelt L, Roses AD, Tjernberg LO, Silberring J, Gandy SE, Winblad B, Greengard P, Nordstedt C, Terenius L (1994). Relative abundance of Alzheimer Aß amyloid peptide variants in Alzheimer disease and normal aging. Proc. Natl. Acad. Sci. USA.

[48] Hartig W, Goldhammer S, Bauer U, Wegner F, Wirths O, Bayer TA, Grosche J (2010). Concomitant detection of ß-amyloid peptides with N-terminal truncation and diffeent C-terminal endings in cortical plaques from cases with Alzheimer´s disease senile monkeys and triple transgenic mice. J. Chem. Neuroanatomy.

[49] Iwatsubo T, Saido TC, Mann DM, Lee VM, Trojanowski JQ (1996). Full-length amyloid beta (1-42(43)) and amino-terminally modified and truncated amyloid-beta 42(43) deposit in diffuse plaques. Am. J. Pathol.

[50] Kuo YM, Emmerling MR, Woods AS, Cotter RJ, Roher AE (1997). Isolation Chemicalm characterization and quantitation of Aß 3-pyroglutamyl peptide from neuritic plaques and vascular amyloid deposits. Biochem. Biophys. Res. Commun.

[51] Mandler M, Rockenstein E, Ubhi K, Hansen L, Adame A, Michael S, Galasko D, Santic R, Mattner F, Masliah E (2012). Detection of peri-synaptic amyloid-b pyroglutamate aggregates in early stages of Alzheimer´s disease and in AbPP transgenic mice using a novel monoclonal antibody. J. Alzheimers Dis.

[52] Bayer TA, Wirths O (2011). Intraneuronal Aß is a trigger for neuronal loss can this be translated into human pathology. Biochem. Soc. Trans.

[53] DeKimpe L, vanHaastert ES, Kaminari A, Zwart R, Rutjes H, Hoozemans J J M, Scheper W (2012). Intracellular accumulation of aggregated pyroglutamate amyloid beta convergence of aging and Aß pathology at the lysosome. AGE DOI 10.1007/s11357-012-9403-0..

[54] Schlenzig D, Ronicke R, Cynis H, Ludwig HH, Scheel E, Reymann K, Saido T, Hause G, Schilling S, Demuth HU (2012). N-terminal pyroglutamate formation of Aß38 and Aß40 enforces oligomer formation and potency to disrupt hippocampal long-term potentiation. J. Neurochem.

[55] Kayed R, Head E, Thompson JL, McIntire TM, Milton SC, Cotman CW, Glabe CG (2003). Common structure of soluble amyloid oligomers implies common mechanism of pathogenesis. Science.

[56] Acero G, Manoutcharian K, Vasilevko V, Munguia M E, Govezensky T, Coronas G, Luz-Madrigal A, Cribas D H, Gevorkian G (2009). Immunodominant epitope and properties of pyroglutamate-modified Aß-specific antibodies produced in rabbits. J. Neuroimmunol.

[57] Kawarabashi T, Younkin L H, Saido T C, Shoji M, Ashe K H, Younkin S G (2001). Age-dependent changes in brain, CSF, and plasma amyloid(beta) protein in the Tg2576 transgenic mouse model of Alzheimer´s disease. J. Neurosci.

[58] Kalback W, Watson M D, Kokjohn T A, Kuo Y M, Weiss N, Luehrs D C, Lopez J, Brune D, Sisodia S S, Staufenbiel M, Emmerling M, Roher A E (2002). APP transgenic mice Tg2576 accumulate Aß peptides that are distinct from the chemically modified and insoluble peptides deposited in Alzheimer’s disease senile plaques. Biochemistry.

[59] Schilling S, Zeitschel U, Hoffmann T, Heiser U, Francke M, Kehlen A, Holzer M, Hutter-Paier B, Prokesch M, Windisch M, Jagla W, Schlenzig D, Lindler C, Rudolph T, Reuter G, Cynis H, Montag D, Demuth HU, Rossner S (2008). Glutaminyl cyclase inhibition attenuates pyroglutamate Aß and Alzheimer´s disease-like pathology. Nat. Med.

[60] Casas C, Sergeant N, Itier JM, Blanchard V, Wirths O, vanderKolk N, Vingtdeux V, vandeSteeg E, Ret G, Canton T, Drobecq H, Clark A, Bonici B, Delacourte A, Benavides J, Schmitz C, Tremp G, Bayer TA, Benoit P, Pradier L (2004). Massive CA1/2 Neuronal Loss with Intraneuronal and N-Terminal Truncated Aß42 Accumulation in a Novel Alzheimer Transgenic Model. Am. J. Pathol.

[61] Bayer TA, Breyhan H, Duan K, Rettig J, Wirths O (2008). Intraneuronal ß-amyloid is a major risk factor novel evidence from the APP/PS1KI mouse model. Neurodegener. Dis.

[62] Breyhan H, Wirths O, Duan K, Marcello A, Rettig J, Bayer TA (2009). APP/PS1KI bigenic mice develop early synaptic deficits and hippocampus atrophy. Acta Neuropathol.

[63] Wirths O, Weis J, Kayed R, Saido TC, Bayer TA (2007). Age-dependent axonal degeneration in an Alzheimer mouse model. Neurobiol. Aging.

[64] Wirths O, Breyhan H, Cynis H, Schilling S, Demuth H U, Bayer T A (2009). Intraneuronal pyroglutamate-Abeta 3-42 triggers neurodegeneration and lethal neurological deficits in a transgenic mouse model. Acta Neuropathol.

[65] Alexandru A, Jagla W, Graubner S, Becker A, Bauscher C, Kohlmann S, Sedlmeier R, Raber KA, Cynis H, Ronicke R, Reymann KG, Petrasch-Parwez E, Hartlage-Rubsamen M, Waniek A, Rossner S, Schilling S, Osmand AP, Demuth HU, vonHorsten S (2011). Selective hippocampal neurodegeneration in transgenic mice expressing small amounts of truncated Ab is induced by pyroglutamate-Aß formation. J. Neurosci.

[66] Jawhar S, Wirths O, Schilling S, Graubner S, Demuth HU, Bayer TA (2011). Overexpression of glutaminyl cyclase, the enzyme responsible for pyroglutamate Ab formation induces behavioral deficits and glutaminyl cyclase knock-out rescues the behavioral phenotype in 5XFAD mice. J. Biol. Chem.

[67] Wittnam JL, Portelius E, Zetterberg H, Gustavsson MK, Schilling S, Koch B, Demuth HU, Blennow K, Wirths O, Bayer TA (2012). Pyroglutamate amyloid ß (Aß) aggravates behavioral deficits in transgenic amyloid mouse model for Alzheimer´s disease. J. Biol. Chem.

[68] Schilling S, Hoffmann T, Manhart S, Hoffmann M, Demuth HU (2004). Glutaminyl cyclases unfold glutamyl cyclase activity under mild acid conditions. FEBS Lett.

[69] Fischer WH, Spiess J (1987). Identification of a mammalian glutaminyl cyclase converting glutaminyl into pyroglutamyl peptides. Proc. Natl. Acad. Sci. USA.

[70] Cynis H, Scheel E, Saido T C, Schilling S, Demuth HU (2008). Amyloidogenic processing of amyloid precursor protein evidence of a pivotal role of glutaminyl cyclase in generation of pyroglutamate-modified amyloid-ß. Biochemistry.

[71] Schilling S, Appl T, Hoffmann T, Cynis H, Schulz K, Jagla W, Friedrich D, Wermann M, Buchholz M, Heiser U, vonHorsten S, Demuth HU (2008). Inhibition of glutaminyl cyclase prevents prevents pGlu-Aß formation after intracortical/hippocampal microinjection in vivo/insitu. J. Neurochem.

[72] DeKimpe L, Bennis A, Zwart R, vanHaastert ES, Hoozemans JJ (2012). Disturbed Ca2+ homeostasis increases glutaminyl cyclase expression connecting two early pathogenic events in Alzheimer´s disease in vitro. PLoS ONE.

[73] Valenti MT, Bolognin S, Zanatta C, Donatelli L, Innamorati G, Pampanin M, Zanusso G, Zatta P, Carbonare LD (2013). Increased glutaminyl cyclase expression in peripheral blood of Alzheimer´s disease patients. J. Alzheimers Dis.

[74] Hartlage-Rubsamen M, Morawski M, Waniek A, Jager C, Zeitschel U, Koch B, Cynis H, Schilling S, Schliebs R, Demuth HU, Rossner S (2011). Glutaminyl cyclase contributes to the formation of focal and diffuse pyroglutamate (pGlu)-Aß deposits in hippocampus via distinct cellular mechanisms. Acta neuropathol.

[75] Cynis H, Schilling S, Bodnar M, Hoffmann T, Heiser U, Saido T C, Demuth H U (2006). Inhibition of glutaminyl cyclase alters pyroglutamate formation in mammalian cells. Biochim. Biophys. Acta.

[76] Schenk D, Barbour R, Dunn W, Gordon G, Grajeda H, Guido T, Hu K, Huang J, Johnson-Wood K, Khan K, Kholodenko D, Lee M, Liao Z, Lieberburg I, Motter R, Mutter L, Soriano F, Shopp G, Vasquez N, Vandevert C, Walker S, Wogulis M, Yednock T, Games D, Seubert P (1999). Immunization with amyloid-ß attenuates Alzheimer-disease-like pathology in the PDAPP mouse. Nature.

[77] Bard F, Cannon C, Barbour R, Burke RL, Games D, Grajeda H, Guido T, Hu K, Huang J, Johnson-Wood K, Khan K, Kholodenko D, Lee M, Lieberburg I, Motter R, Nguyen M, Soriano F, Vasquez N, Weiss K, Welch B, Seubert P, Schenk D, Yednock T (2000). Peripherally administered antibodies against amyloid-ß peptide enter the central nervous system and reduce pathology in a mouse model of Alzheimer’s disease. Nat. Med.

[78] Lemere CA, Beierschmitt A, Iglesias M, Spooner ET, Bloom JK, Leverone JF, Zheng JB, Seabrook TJ, Louard D, Li D, Selkoe DJ, Palmour RM, Ervin FR (2004). Alzheimer’s disease Aß vaccine reduces central nervous system Aß levels in a non-human primate, the Caribbean vervet. Am. J. Pathol.

[79] Wilcock DM, Rojiani A, Rosenthal A, Levkowitz G, Subbarao S, Alamed J, Wilson D, Wilson N, Freeman M J, Gordon M N, Morgan D (2004). Passive amyloid immunotherapy clears amyloid and transiently activates microglia in a transgenic mouse model of amyloid deposition. J. Neurosci.

[80] Brody D L, Holtzman D M (2008). Active and passive immunotherapy for neurodegenerative disorders. Annu. Rev. Neurosci.

[81] Head E, Pop V, Vasilevko V, Hill. M, Saing T, Sarsoza F, Nistor M, Christie LA, Milton S, Glabe C, Barrett E, Cribbs D (2008). A two-year study with fibrillar beta-amyloid (Abeta) immunization in aged canines effects on cognitive function and brain Abeta. J. Neurosci.

[82] Biscaro B, Lindvall O, Hock C, Ekdahl C T, Nitsch R M (2009). Aß immunotherapy protects morphology and survival of adult-born neurons in doubly transgenic APP/PS1 mice. J. Neurosci.

[83] Morgan D (2011). Immunotherapy for Alzheimer´s disease. J. Intern. Med.

[84] Lemere C A, Masliah E (2010). Can Alzheimer disease be prevented by amyloid-ß immunotherapy. Nat. Rev. Neurol.

[85] Roher AE, Maarouf CL, Daugs ID, Kokjohn TA, Hunter JM, Sabbagh MN, Beach TG (2011). Neuropathology and amyloid-b spectrum in a bapineuzumab immunotherapy recipient. J. Alzheimers Dis.

[86] Bacskai BJ, Kajdasz ST, McLellan M E, Games D, Seubert P, Schenk D, Hyman B T (2002). Non-Fc-mediated mechanisms are involved in clearance of amyloid-beta in vivo by immunotherapy. J. Neurosci.

[87] Zago W, Buttini M, Comery T A, Nishioka C, Gardai S J, Seubert P, Games D, Bard F, Schenk D, Kinney G G (2012). Neutralization of soluble synaptotoxic amyloid ß species by antibodies is epitope speicifc. J. Neurosci.

[88] Perez-Garmendia R, Ibarra-Bracamontes V, Vasilevko V, Luna-Munoz J, Mena R, Govezensky T, Acero G, Manoutcharian K, Cribs D H, Gevorkian G (2010). Anti-11 [E]-pyroglutamate-modified amyloid ß antibodies cross-react with other pathological Aß species relevante for immunotherapy. J. Neuroimmunol.

[89] Venkataramani V, Wirths O, Budka H, Hartig W, Kovacs G G, Bayer T A (2012). Antibody 9D5 recognizes oligomeric pyroglutamate amyloid-b in a fraction of amyloid-b deposits in Alzheimer´s disease without cross-reactivity with other protein aggregates. J. Alzheimers Dis.

[90] Frost J L, Liu B, Kleinschmidt M, Schilling S, Demuth H U, Lemere C A (2012). Passive immunization against pyroglutamate-3 amyloid-b reduces plaque burden in Alzheimer-like transgenic mice a pilot study. Neurodegenerative Dis.

[91] DeMattos R, Lu J, Tang Y, Racke M M, DeLong C A, Tzaferis J A, Hole J T, Forster B M, McDonnell P C, Liu F, Kinley R D, Jordan WH, Hutton M L (2012). A plaque-specific antibody clears existing ß-amyloid plaques in Alzheimer´s disease mice. Neuron.

[92] Tomljenovic L, Shaw CA (2011). Aluminum vaccine adjuvants are they safe. Curr. Med. Chem.

[93] Siemers E R, Friedrich S, Dean R A, Gonzalez C R, Farlow M R, Paul S M, DeMattos R B (2010). Safety and changes in plasma and cerebrospinal fluid amyloid beta after a single administration of an amyloid beta monoclonal antibody in subjects with Alzheimer disease. Clin. Neuropharmacol.

[94] Samadi H, Sultzer D (2011). Solanezumab for Alzheimer´s disease. Expert Opin. Biol. Ther.

[95] Farlow M, Arnold S E, vanDyck C H, Aisen P S, Snider B J, Porsteinsson A P, Friedrich S, Dean R A, Gonzalez C, Sethuraman G, DeMattos R B, Mohs R, Paul S M, Siemers E R (2012). Safety and biomarker effects of solanezumab in patients with Alzheimer´s disease. Alzheimers Dement.

[96] Imbimbo B P, Ottonello S, Frisardi V, Solfrizzi V, Greco A, Seripa D, Pilotto A, Panza F (2012). Solanezumab for the treatment of mild-to-moderate Alzheimer´s disease. Expert Rev. Clin. Immunol.

